# A smart sealed nucleic acid biosensor based on endogenous reference gene detection to screen and identify mammals on site

**DOI:** 10.1038/srep43453

**Published:** 2017-02-24

**Authors:** Yuancong Xu, Wenjin Xiang, Qin Wang, Nan Cheng, Li Zhang, Kunlun Huang, Wentao Xu

**Affiliations:** 1 Beijing Advanced Innovation Center for Food Nutrition and Human Health, College of Food Science & Nutritional Engineering, China Agricultural University, Beijing, 100083, China; 2 Laboratory of Food Safety, College of Food Science and Nutritional Engineering, China Agricultural University, Beijing 100083, China; 3 Institute of Animal Quarantine, Chinese Academy of Inspection and Quarantine, Beijing 100123, China

## Abstract

The identification of meat adulteration is a hotspot for food research worldwide. In this paper, a smart and sealed biosensor that combines loop-mediated isothermal amplification (LAMP) with a lateral flow device (LFD) was developed, resulting in the universal mammalian assessment on site. First, the highly specific chromosomal Glucagon gene (*Gcg*) was chosen as the endogenous reference gene, and the LAMP approach provided double-labeled duplex DNA products using FITC- and BIO- modified primers. Then, an LFD strategy was used for specific signal recognition through an immunoassay. Meanwhile, LFD-LAMP was compared to LAMP and real-time LAMP, the results showed consistent high specificity and sensitivity but in a more convenient and easy-to-use system. In addition, the detection limit was as low as 10 pg, which was equivalent to 3~5 copies in mammals. All of the reactions were performed in a sealed system regardless of the amplification process or products recognized. Therefore, the smart design demonstrated significantly high specificity and the ability to detect trace amounts of DNA in complex and processed foods with mammalian meat. As a universal and specific platform for the detection of mammalian DNA, this smart biosensor is an excellent prospect for species identification and meat adulteration.

The consumption of mammalian meat and processed products constitutes a significant apportion of the total meat consumed. The adulteration of meat with cheaper meats and vegetable protein has always created controversy in the expensing process. On the one hand, with the rapid development of agriculture and urbanization over the past twenty years, the authenticity and safety of meat have been concerns for many consumers^1^. This is because fraudulent consumption is related to public health, religious factors, wholesomeness and unhealthy competition in the meat market^2^. On the other hand, red meat is an integral part of the balanced diet for many adults and is the major source of docosapentaenoic acid (DPA), which accumulates in mammals^3^. With red meat consumption, the risk of colorectal cancer increases due to the ingestion of *N-*nitrosation, although other nutrients, including vitamin B6, vitamin B12, and calcium, could protect against colorectal carcinogenesis^4^,^5^. Furthermore, red and processed meats are associated with a higher incidence of coronary heart disease^6^,^7^. Therefore, it is of vital importance to accurately and rapidly identify the different species of mammals or red meat within processed meats, which would greatly benefit consumers by suppressing adulteration in the meat industry. In addition, the species identification of mammals will contribute to the screening and identification of wild animals. Therefore, it is critical to accurately and rapidly identify the mammalian species or the red meat components in meat products.

Various methods have been used to detect species of adulterated meat. For example, immunological procedures^8^, sensory evaluation^9^, spectroscopic methods^10^, and molecular biology techniques^11^ have been employed to analyze meat. Most biotechnologies displayed strong specificity and high sensitivity, except for the protein-based assay, which was not sensitive enough to differentiate closely related species due to cross-reactivity^12^. Therefore, DNA has always been chosen as the target for molecular detection due to its stability at high temperature, availability and conserved structure within all organisms in a species^11^. Species-specific DNA fragments have been widely utilized by polymerase chain reaction (PCR) and real-time PCR to rapidly and precisely identify the original species in processed meat and meat products^13^,^14^. Subsequently, a common primer multiplex-PCR (CP-M-PCR) assay had been used for the simultaneous identification of four meat species and their products^15^. Other correlation techniques, including random amplification of polymorphic DNA (RAPD), quantitative competitive PCR, microsatellites, PCR-based amplified fragment length polymorphism (AFLP), nested primer PCR and loop-mediated isothermal amplification (LAMP), have also been used to identify meat species^16^. The LAMP method, which uses self-recurring strand-displacement DNA synthesis, was developed as an alternative method capable of detecting very low quantities of target nucleic acid under isothermal conditions^17^. Because LAMP can be performed using an inexpensive heating block or water bath, thereby removing dependence upon stable electricity and allowing LAMP to be suitable for on-site detection. Moreover, the products of this assay could be identified using a lateral flow device (LFD) in sealed plastic devices to avoid any possible cross contamination^18^. Consequently, the LFD-LAMP technique is a reliable, pollution-free and powerful tool for detecting meat adulterations because of its extraordinary capacity for detecting trace amounts of DNA.

Endogenous reference genes are widely used in ingredient identification and analysis. During food production, many unscrupulous merchants commit food fraud to increase profit, thereby deceiving consumers. Consequently, using endogenous reference genes to identify food adulteration is a smart choice. A reliable endogenous reference gene should have high species-specificity, a low constant copy number and low heterogeneity in different cultivars. Low copy genes generally have less variation; therefore, a single-copy gene is often the best choice for endogenous reference genes^19^. Qualitative and quantitative approaches using endogenous reference genes can be employed to evaluate the quality of the DNA sample, analyze the food source to determine food allergens and determine the ingredients in complex food admixture^20^. Numerous studies have reported the identification of mammalian species in various animal taxa using DNA-based methods. The mitochondrial cytochrome b (mt cytb) gene has been used to identify marine mammal samples and processed products by analyzing the amplicons resulting from random amplification of polymorphic DNA (RAPD) and single-strand conformational polymorphism (SSCP)^21^. Due to a large number of mismatches in *cyt b* sequences, a broad-spectrum molecular assay with degenerated primers was designed to authenticate diverse small mammal species^22^. In addition, a total of 28 different mammals were tested for species-specific molecular markers by the PCR and pyrosequencing analysis of two short mitochondrial DNA amplicons obtained by different primers for 12 S rRNA and 16 S rRNA. The drawback of this reference gene was the existence of mutations and single nucleotide polymorphisms (SNPs) in the ribosomal gene^23^. Although mitochondrial genes have been used in many studies for species identification, they are still not good endogenous reference genes and are unsuitable for quantitative analyses due to high copy numbers.

In this study, a smart sealed biosensor using loop-mediated isothermal amplification and a lateral flow device was developed to identify mammals for the first time. First, in the LAMP approach, FITC- and BIO-labeled products were amplified by modified primers. Then, the double-labelled products from mammals were captured on the test line of LFD through an immunoassay (Fig. 1). To ensure the accuracy of the biosensor, the highly specific chromosomal gene Glucagon (*Gcg*) was selected as the endogenous reference gene. Southern blot was used to confirm that the *Gcg* gene exists in a single copy in the mammalian genome. Moreover, LAMP was selected for its specificity and sensitivity due to its extraordinary capacity for detecting damaged DNA from deeply processed meat products. The limitation was as low as 10 pg of DNA, which is equivalent to 4 copies; therefore, this approach was highly sensitive in screening for mammalian DNA. For on-site detection, it is critical to use smart instruments that are lightweight. In addition, the developed nucleic acid biosensor is well suited for the detection of mammalian DNA quickly with small sizes and high agility. In conclusion, this study describes a convenient and accurate biosensor for the detection of mammalian species, and its rapidity and utility make this biosensor more suitable for on-site detection.

## Results and Discussion

### Selection of the mammalian endogenous references gene

**Figure 2:**
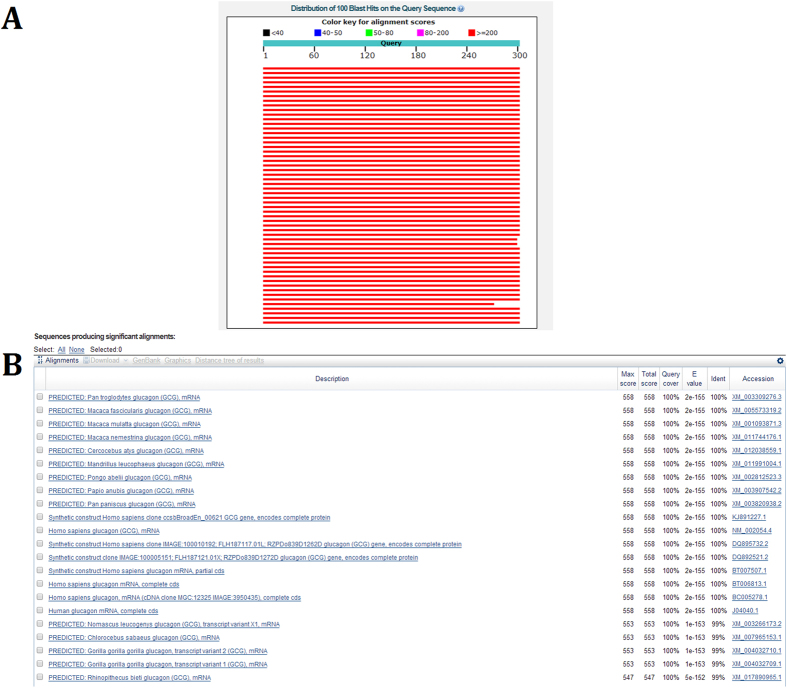
The BLAST results of the *Gcg* gene.

The chromosomal *Gcg* gene encodes glucagon, a twenty-nine-amino acid polypeptide hormone^24^. Its chief function is to stimulate glycogenolysis, gluconeogenesis and ketogenesis in the liver. In addition, biosynthetic studies have demonstrated the importance of glucagon in mammals^25^. An appropriate endogenous references gene is necessary to ensure the detection of meat species more precisely and practically. The obtained 22 Gcg sequences from the NCBI were analyzed by DNAMAN (Figure S1), and then the conserved sequence (302 bp) was analyzed by BLAST (basic local alignment search tool) with all known Gcg sequences to confirm that the mammalian *Gcg* gene was species specific. The BLAST alignment analysis is illustrated in Fig. 2, which shows that the 302 bp sequence for the *Gcg* gene exhibited high homogeneity in the mammalian species and lower homogeneity with other species. At the same time, the LAMP method was well known that had powerful capable to detect very low quantities of DNA and typically amplicons would be about 200 bp^17^. Consequently, the 302 bp fragment was well suited for primer design and species-specific detection. Specific primers for G-FIP/BIP and G-F3/B3 were used to detect the *Gcg* gene in qualitative and quantitative LAMP.

### Specificity and allelic variation of the Gcg gene among mammals by qualitative and quantitative LAMP assays

**Figure 3:**
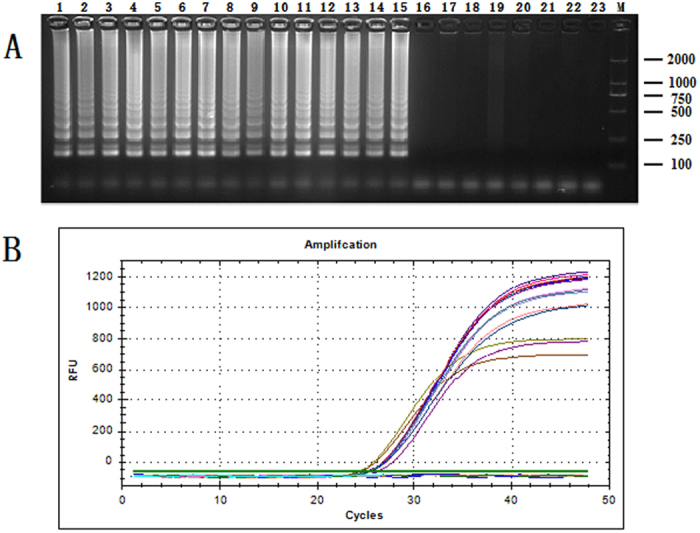
The specificity detection with qualitative (**A**) and quantitative (**B**) LAMP. Lane M: DNA Marker DL 2000. Lane 1, pork; 2, beef; 3, sheep; 4, horse; 5, donkey; 6, mouse; 7, dog; 8, rabbit; 9, water buffalo; 10, camel; 11, sable; 12, deer; 13, yak; 14, monkey; 15, human placenta; 16, chicken; 17, duck; 18, goose; 19, fish; 20, trionyx; 21, bullfrog; 22, sparrow; 23, negative control.

A reliable endogenous reference gene should have high species specificity in different varieties and should have no allelic variation within the same variety^26^. The genomic DNA of 22 species animals (Figure S2), including 15 mammalian species and 7 other meat varieties, was used to determine the specificity and allelic variation of the *Gcg* gene by qualitative and quantitative LAMP assays using the primers G-FIP/BIP and G-F3/B3. The products were analyzed by 2% agarose gel electrophoresis (Fig. 3A). Positive reactions were observed for all mammals, whereas no amplified products were observed for the other meat varieties. All qualitative LAMP amplicons of identical size were obtained for all tested mammalian varieties, which demonstrated that there were no major sequence differences between the 15 mammals. The recovered products should be sequenced to make sure the conservative^20^, and the sequences of the fifteen products were identical. The qualitative results were consistent with the results that we obtained from the quantitative LAMP approach, exhibiting fluorescent signals only in the mammalian samples and not the other tested species (Fig. 3B). In addition, the quantitative LAMP analyses performed with the DNA extracted from the mammalian varieties produced similar Ct values ranging from 24.83 to 26.69. Therefore, these results indicated that the *Gcg* gene was highly specific and had no allelic variation in mammals.

### Copy number verification by Southern blot

**Figure 4:**
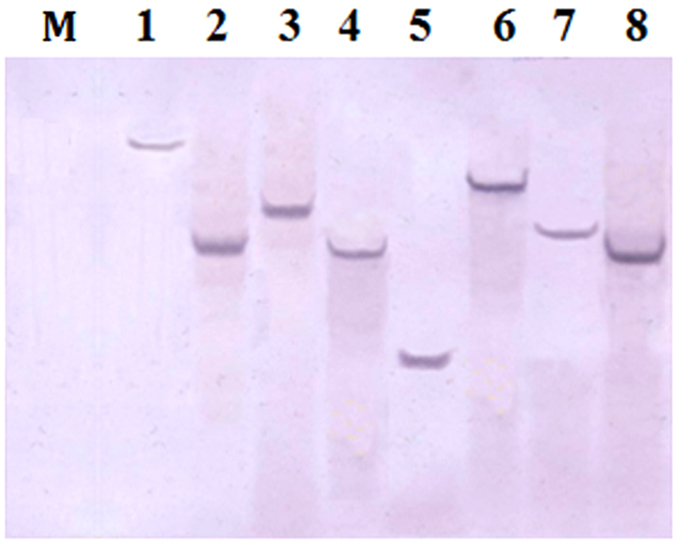
The southern blot result for the *Gcg* copy number of 8 mammalian species. Lane 1, pork DNA digested by BamHI; 2, beef DNA digested by BamHI; 3, sheep DNA digested by BamHI; 4, horse DNA digested by BamHI; 5, dog DNA digested by BamHI; 6, mouse DNA digested by BamHI; 7, rabbit DNA digested by BamHI; 8, human placenta DNA digested by BamHI; Lane M, Marker (not recognized).

A mammalian endogenous reference gene sequence should be highly conserved with low copy number throughout all mammalian species to be broadly applicable^19^. A group previously described the *Gcg* gene as highly conserved between species, and the *Gcg* gene of porcine species has been widely used as single-copy control region to determine the copy number of a target gene^27^,^28^. Concurrently, analysis using the DNAMAN software revealed that the 302 bp sequence of the *Gcg* gene was extraordinarily conserved in mammalian species and significantly different from that in other species (Figure S1). Preliminary analysis of the copy number of that particular sequence in 8 mammals), including pork (*Sus scrofa*), beef (*Bos taurus*), sheep (*Ovis aries*), horse (*Equus caballus*), dog (*Canis lupus familiaris*), mouse (*Mus musculus*), rabbit (*Oryctolagus cuniculus*) and human (*Homo sapiens*), was obtained through Ensembl (http://www.ensembl.org). The results showed that this particular *Gcg* gene had a single copy in these mammalian species (data not shown). Moreover, southern blot is regarded as the gold standard for determining the copy numbers of target genes. Hence, southern blot was used to confirm the *Gcg* copy number obtained from Ensembl. Southern blot analysis on the selected partial *Gcg* sequence revealed a single band from the *BamHI*-digested DNA (Fig. 4), suggesting that one copy was present in each of the 8 tested mammals. This result indicated that the *Gcg* gene was present in a single copy in the mammalian genome. In addition, it could also be used as a control region to determine the copy number of a target gene.

### Sensitivity of the quantitative LAMP assay

**Figure 5:**
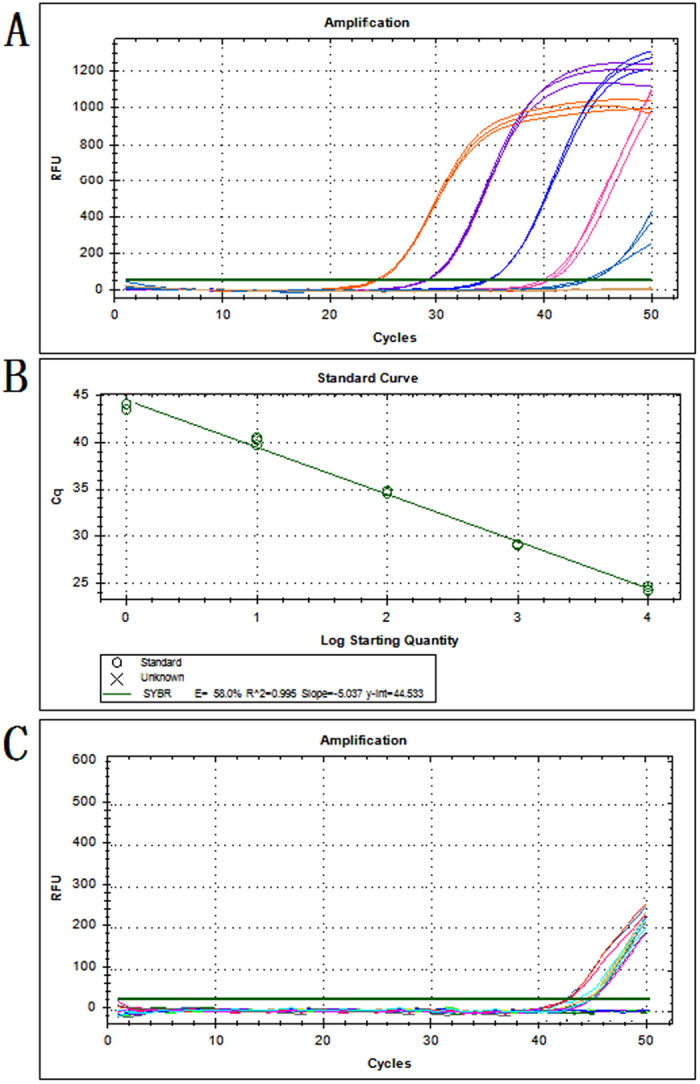
The sensitivity of *Gcg* gene detection in quantitative LAMP. (**A**) The amplification plots generated by 10-fold serial dilutions of cow genomic DNA ranging from 10^5^ pg to 1 pg. (**B**) The standard curve generated from the amplification plots. (**C**) The amplification plots generated by genomic DNA from 15 different mammalian species ranging from 10 pg to 1 pg.

Cow is the classic mammal, and beef is the classic red meat. Therefore, the sensitivity of the quantitative detection of the *Gcg* endogenous reference gene was evaluated with a series of real-time LAMP assays using 10-fold serial dilutions of beef genomic DNA that ranged from 100 ng to 1 pg. This assay showed that the LAMP method exhibited high sensitivity. The amplified curves generated by monitoring the fluorescence signal are shown in Fig. 5A. In addition, the amplified fluorescent signals were detectable even when the DNA template quantities were as low as 10 pg. The genome of *Bos taurus* has been sequenced and assembled in the NCBI database, and the total length is 2,670,139,648 bp (GenBank assembly accession: GCA_000003055.5). Based on a previous study, one picogram (pg) is equal to 965 million base pairs (Mbp)^29^. Hence, the limit is approximately equivalent to 4 copies according to the *Bos taurus* genomic DNA calculation of 2.767 pg/copy.

A standard curve of the *Gcg* gene using the quantitative LAMP system was constructed to evaluate the accuracy of the quantification system for relative quantitative analyses. As illustrated in Fig. 5B, there is a linear relationship between the log of the DNA concentration of the initial template DNA and the Ct value, with a satisfactory correlation coefficient R^2^ (0.995) and a slope of −5.037. This result demonstrated that the tested beef DNA quantities and the corresponding Ct values were highly correlated; this may potentially apply to other mammals as well. The sensitivity of quantitative LAMP analyses of 14 other mammals was analyzed using their respective genomic DNA (10 pg and 1 pg). Figure 5C illustrated the lowest detectable amplified fluorescent signals of 10 pg, identical to the beef analysis. According to previous literature, there was no limit of quantitative detection due to the mitochondrial gene with high copies^21^,^22^,^30^. In addition, the limitation of the quantitative LAMP assay in 15 mammalian species was 3~5 copies (data shown in Table S3), further demonstrating that the *Gcg* gene could be used as an endogenous reference gene for accurate quantitative detection in mammals.

### Application of the LFD-LAMP assay in meat detection

**Figure 6:**
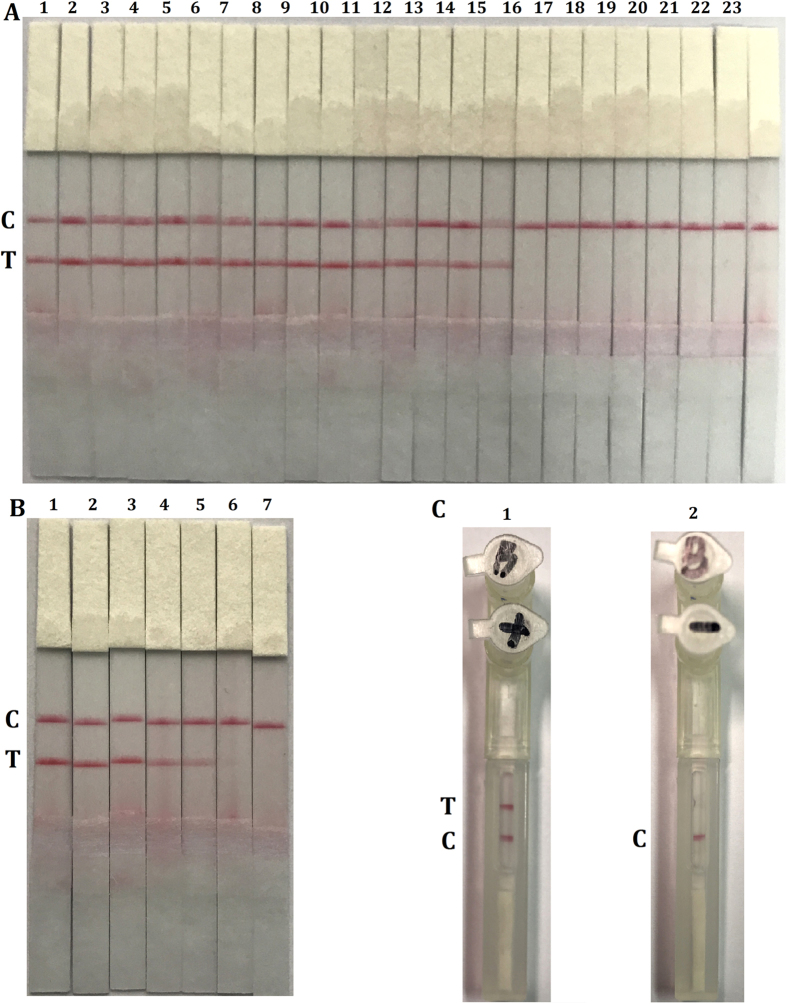
Analysis of the LAMP products with the LFD and portable sealed biosensor. (**A**) Specificity test of the LFD-LAMP assay with LFD. Lane 1, pork; 2, beef; 3, sheep; 4, horse; 5, donkey; 6, mouse; 7, dog; 8, rabbit; 9, water buffalo; 10, camel; 11, sable; 12, deer; 13, yak; 14, monkey; 15, human placenta; 16, chicken; 17, duck; 18, goose; 19, fish; 20, trionyx; 21, bullfrog; 22, sparrow; 23, negative control. (**B**) Sensitivity test of the LFD-LAMP products by LFD. Lane 1, 100 ng; 2, 10 ng; 3, 1 ng; 4, 0.1 ng; 5, 10 pg; 6, 1 pg; 7, negative control. (**C**) LAMP positive and negative reactions were subjected to portable sealed biosensor visual detection. Lane 1, positive control; 2, negative control. All pictures were collected after 3 min.

Recently, both amplification methods and product detection technologies have been constantly modified and widely used in many areas. For the identification of amplicons, agarose gel electrophoresis, turbidity measurements and visual inspection of color changes all have inherent limitations involving time, cross-contamination and instrument-dependence^31^. For greater convenience and accuracy, the LFD-LAMP assay^32^ was used to detect the *Gcg* gene. To ensure the feasibility of the visual strategy, the FITC- and BIO-labeled primers were used to detect the presence of the mammalian *Gcg* gene, which different from the previous report with a modified primer and a labelled probe^33^. Considering the data from Fig. 6A and B, the detection specificity and sensitivity of the LFD-LAMP method proved to be in accordance with those of qualitative and quantitative LAMP, respectively.

Moreover, a portable sealed nucleic acid biosensor was obtained when the paper-based LFD was embedded in a smart plastic device. The performance evaluation of the biosensor showed that the system had good recognition and stability (Fig. 6C). To measure the utility of the aforementioned biosensor, various meat materials and processed foods were analyzed with the LFD-LAMP approach. The practical detection results (Table 1) demonstrated that the mammalian elements of only fast food (McDonald’s and Subway), meat products (Dried Pork Slice, Pork Flake Rolls and Lamb Rolls), and mixed meats could be accurately identified. The detection results of other foods were all negative. This means that there were no mammalian components in the kinds of feed or food that were produced by adding flavors and fragrances. In other words, the developed biosensor demonstrated significantly high specificity and the ability to detect trace amounts of DNA in complex and processed foods with mammalian meat.

Considering the data from the LAMP and LFD-LAMP assays, we conclude that the *Gcg* gene was an effective and accurate endogenous reference gene for the detection of mammalian material in foods, including highly processed foods. In addition, the approach we developed can also be an accurate way to identify the specific species in processed products. It has shown great promise for the specific species identification in known meat products.

## Conclusions

The adulteration of meat is a serious problem to limit the development of the industry and connect with the public health, religious factors, wholesomeness and unhealthy competition in meat market. It is critically important to accurately and rapidly identify the mammalian species or the red meat components in products.

In the present study, a simple, accurate and rapid testing biosensor was established for detection of mammals in practical. Due to DNA was damaged in most deeply processed, many molecular technologies had no capable to analyze the meat components accurately. However, LFD-LAMP technique can be a reliable, pollution-free and represents a powerful detection tool for meat adulterations, because the inordinate capacity for detecting trace amounts of DNA. In our strategy, two primers were all labeled instead of the extra probe mentioned in common LFD-LAMP assay, and the limitation was as low as 10 pg of DNA, which indicates that this method was sufficiently sensitive to identify mammals. The smart closed nucleic acid biosensor requires make this technique suitable as an on-site tool in locations where more complex laboratory equipment is not available.

Moreover, the specificity of the biosensor for practical detection was of crucial importance. The chromosomal gene *Gcg* has been selected as the universal endogenous reference gene. All data of the specificity, allelic validation, copy number and sensitivity demonstrated that the *Gcg* gene is very satisfied with the detection of mammalian ingredients in unknown meat products as an endogenous reference gene. At the same time, the *Gcg* gene also could be a specific endogenous reference gene to identify particular mammals. It showed great promise for the further species identification in known meat products. And it also could be a control region to determine the copy number of measured gene.

Therefore, the amazing combination of the LFD-LAMP and the *Gcg* gene provides great convenient for the fast detection of the sources of meats. The developed biosensor demonstrated significantly high specific and ability to detect trace amounts of DNA in complexed and processed mammals. In addition, the approach we developed also can be a specific screening method for an accurate way to identify the known species in processed products and species identification. As a universal and specific platform for the detection of mammals, this smart biosensor makes excellent prospects for the species identification and meat adulteration.

## Materials and Methods

### Samples

Samples from a total of 22 animal species (Table S1), including pork (*Sus scrofa*), beef (*Bos taurus*), sheep (*Ovis aries*), dog (*Canis lupus familiaris*), rabbit (*Oryctolagus cuniculus*), yak (*Bos mutus*), horse (*Equus caballus*), donkey (*Equus asinus*), mouse (*Mus musculus*), monkey (*Macaca fascicularis*), chicken (*Gallus gallus*), duck (*Anas platyrhynchos*), goose (*Goose calicivirus*), yellow croaker (*Pseudosciaena polyactis*), trionyx (*Trionyx sinensis*), bullfrog (*Rana catesbeiana*), and sparrow (*Passer montanus),* were obtained from the Institute of Zoology, Chinese Academy of Sciences. Samples from water buffalo (*Bubalus bubalis*), sable (*Martes zibellina*), camel (*Camelus bactrianus*), and deer (*Cervus nippon*) were kindly provided by Dr. Zongmeng Li from the Tianjin Entry-Exit Inspection Bureau. A sample of human placenta (*Homo sapiens*) was provided by the Air Force General Hospital.

All the animals were fed the same standard diet and raised under the same conditions. All of the protocols involving the use of animals were approved by the Institutional Animal Care and Use Committee (IACUC) at Institute of Animal Sciences, Chinese Academy of Agricultural Sciences. All experiments were performed in accordance with the approved guidelines for animal care and management of research projects.

### DNA analysis and extraction

The obtained 18 Gcg sequences from NCBI were all analyzed by DNAman V6. Software. Then the conservative fragment was evaluated with BLAST (Figure S1). The DNA samples used for detection were extracted using the TIANamp Genomic DNA Kit (Tiangen, Beijing, China) according to the manufacturer’s specifications. The DNA quality was analyzed by electrophoresis on a 1.0% agarose gel (Figure S2). The DNA solution was stored at 4 °C and used as DNA templates in subsequent analyses.

### Primer design

The *Gcg* gene was chosen as the endogenous reference gene for mammals. In addition, the sequence data of different species were obtained from GenBank (Table S1). The isothermal primers were designed using Primer Explorer V5 software from Eiken Chemical Co. Ltd., Japan (https://primerexplorer.jp/lampv5/index.html) (Figure S3), and the others were designed using Primer 5.0. As shown in Table S2, the G-FIP/BIP and G-F3/B3 primers were used to analyze the *Gcg* gene for qualitative and quantitative LAMP. For the LFD-LAMP strategy, G-FIP and G-BIP primers were labeled by fluorescein isothiocyanate (FITC) and biotin (BIO) at their 5′ end, respectively. The forward inner primer (G-FIP) contained a sequence complementary to F1 (F1c) followed by a T-T-T-T linker and F2; the reverse inner primer (G-BIP) contained a sequence complementary to B1 (B1c) followed by a T-T-T-T linker and B2. The two LAMP outer primers, G-F3 and G-B3, were outside of the G-FIP and G-BIP regions, respectively. Primers for G-SF/SR were designed to analyze the copy number by Southern blot. All of the primers were synthesized by Invitrogen Trading (Shanghai) Co., Ltd. (Beijing, China).

### DNA amplification by LAMP

Qualitative LAMP amplification was carried out on a Bio-Rad S1000^TM^ thermal cycler (BIO-RAD, USA). Each reaction was carried out in a 25 μL reaction mixture: 1x ThermoPol buffer, 1.6 Μm inner primers (G-FIP and G-BIP), 0.2 μM outer primers (G-F3 and G-B3), 0.4 mM dNTPs, 1.0 M betaine, 5 mM MgSO_4_, 8 U of Bst polymerase large fragments (New England Biolabs Ltd., Beijing, China) and 2 μL of DNA template. The mixture was incubated at 64 °C for 40 min, and the reaction was terminated by heating at 85 °C for 5 min. The amplified products were analyzed by 2% agarose gel electrophoresis. Three biological replicates were conducted, and each biological replicate was analyzed.

Quantitative LAMP analysis was performed on a CFX96^TM^ Real-Time System instrument (BIO-RAD, USA). The specificity and sensitivity were measured in a 25 μL reaction mixture containing 1x ThermoPol buffer, 1.6 Μm inner primers (G-FIP and G-BIP), 0.2 μM outer primers (G-F3 and G-B3), 0.4 mM dNTPs, 1.0 M betaine, 5 mM MgSO_4_, 8 U of Bst polymerase large fragment (New England Biolabs Ltd., Beijing, China), 0.8x EVA Green (Biotium, USA)^31^ and 2 μL of DNA template. A total of 10^5^ pg to 1 pg of DNA was used per reaction (10-fold serial dilutions). The reactions cycled with the following program: 5 min at 65 °C and 40 cycles of 1 min at 65 °C, followed by melting curve analysis. Each sample was quantified in triplicate for each biological replicate, and three biological replicates were analyzed.

### Southern blot

DNA samples (30 mg) from eight mammalian varieties were completely digested with BamHI. Next, the digested samples were resolved with 0.8% agarose gel electrophoresis at a constant voltage of 40 V for 4–5 h and then transferred onto a nylon membrane. A 302 bp DNA fragment amplified by the primer G-SF/SR was used as the hybridization probe. This DNA fragment was labeled with α-[32 P]-dCTP using a DNA Labeling kit. Hybridization was performed at 62 °C for 10 h, and the filter was washed at room temperature with 2 × SSC/0.1% SDS and 1 × SSC/0.1% SDS for 10 min each and at 60 °C with 0.2 × SSC/0.1% SDS for 30 min. Then, P-labeled probe was added to the hybridization bag, which was then incubated overnight at 65 °C. The washing and detection steps were performed according to the instructions in the kit. Three biological replicates were conducted, and two technical replicates were analyzed for each biological replicate.

### LFD-LAMP strategy

Au-NPs were prepared in a 500 mL solution. For this, 4 mL of 1% trisodium citrate was added to 96 mL of boiling 0.01% HAuCl_4_, stirred for 30 min, and then cooled to room temperature. Then, 1 mL of a 10-fold-concentrated Au-NP solution was mixed with 5 μL of 10 mM anti-biotin antibody and incubated at room temperature for 1 h, after which 20 μL of 10% BSA and 5 μL of 10% NaCl were added, gently mixed for 2 h, and centrifuged for 20 min at 12,000 rpm. Then, the red precipitate of anti-biotin-Au-NP conjugates was sprayed onto the conjugate pad. The NC membrane was dried at 37 °C for 12 h and then modified with 1.0 mg mL^−1^ FITC antibodies and goat anti-mouse IgG to form the test and control lines, respectively. The completed LFD biosensor was assembled with four parts on a plastic backing, including the sample pad, the conjugate pad, the NC membranes and the absorption pad from bottom to top.

In the LFD-LAMP strategy, FITC- and BIO-labeled inner primers were used to obtain the products. For this, 50 μL of running buffer containing 4 × SSC, 2% BSA and 0.05% Tween-20 (pH 7.0) was mixed with the labeled products. The sample pad of the LFD biosensor was dipped into the mixture, and the lines were visualized within 5 min.

## Additional Information

**How to cite this article**: Xu, Y. *et al*. A smart sealed nucleic acid biosensor based on endogenous reference gene detection to screen and identify mammals on site. *Sci. Rep.* **7**, 43453; doi: 10.1038/srep43453 (2017).

**Publisher's note:** Springer Nature remains neutral with regard to jurisdictional claims in published maps and institutional affiliations.

## Supplementary Material

Supplementary Material

## Figures and Tables

**Figure 1:**
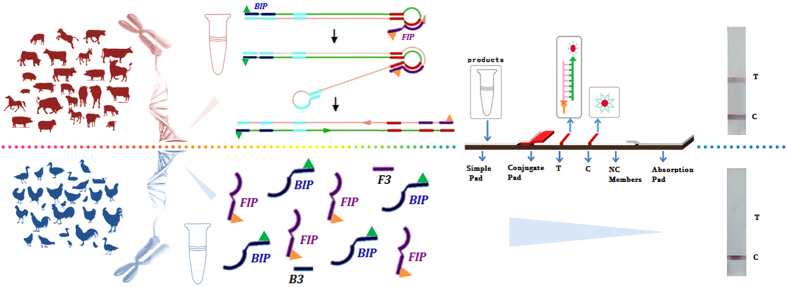
Schematic illustration of the design of the mammalian biosensor system. The approach combines the amplification features of LAMP with the visual detection on a LFD to screen and identify mammals or red meat within processed meats rapidly. Generally, mammalian include the red meat, so that the mammalian was drawn in red with pictorial diagram. By contrast, other species was presented in dark blue diagram. Genomic DNA was analyzed to seek the endogenous reference gene, and used to determine the specificity, allelic variation and sensitivity by LAMP. For LAMP approach, in the presence of mammalian, FITC- and BIO-labeled products were obtained by modified primers. And there were only free labelled-primers in the closed tube in absence of mammalian. For LFD strategy, Anti-biotin-labeled Au-NPs, anti-FITC antibodies and secondary antibody were pre-immobilized on the conjugate pad, test line (T) and control line (C) of the LFD, respectively. Then, the products with running buffer were applied on the sample application pad of LFD. Typically, when the solution with double-labelled products migrates by capillary action and passes the conjugate pad, the biotin on the products and primers reacted with the anti-biotin on the Au-NP surface to form a DNA-biotin–anti-biotin–Au-NP complex and continued to migrate along the strip. Then the positive complexes were captured on the test line by the immunoreactions between anti-FITC on the test line and the FITC in the positive complexes. The accumulation of Au-NPs is visualized as a characteristic red band on test line. The excess anti-biotin–Au-NPs continued to move and were captured on the control line by immunoreactions between the secondary antibody and anti-biotin on the Au-NPs, forming another red band. In the absence of mammalian, there was no dual-labeled product in the solution; therefore, no anti-biotin–Au-NP conjugate was captured on the test line of the LFD, and no red band was observed on the test line. For this method, a red band on the control line showed that the LFD was working properly.

**Table 1:** Detection results of mixed meat materials and processed products.

Samples	Origin	Testing results
McDonald’s Big Mac	McDonald’s of China	+
Subway’s Beef Sandwich	Subway of China	+
Shin Ramyun with Beef and Mushroom flavor	Nongshim of Shanghai	−
Three Squirrels’ dried pork slice	Three Squirrels of Anhui	+
Xiangji’s Pork Flake Rolls	Macao	+
Little sister jun-ya zhang grilled BBQ snack noodles	Taiwan	−
MACKIE’s chips with Bacon flavor	England	−
JIUR’s Potatoes Wedges with barbecue flavor	Korea	−
Cattle and sheep feed	CP FEED	−
Quick-frozen Lamb Rolls	Inner Mongolia	+
Mixed meats (10% pork, 50% duck, 40% chicken)	This study	+
Mixed meats (5% beef, 5% pork, 40% chicken)	This study	+
Mixed meats (1% sheep, 59% duck, 40% chicken)	This study	+
Mixed meats (0.5% horse, 59.5% duck, 40% chicken)	This study	+
Mixed meats (0.1% beef, 59.9% duck, 40% chicken)	This study	+
Mixed meats (0.01% mouse, 59.99% duck, 40% chicken)	This study	+
