# Supplementary material for: A smart sealed nucleic acid biosensor based on endogenous reference gene detection to screen and identify mammals on site
Source: Sci Rep. 2017 Feb 24;7:43453. doi: 10.1038/srep43453 (PMC5324065; doi:10.1038/srep43453)
Supplement: Supplementary Material [file srep43453-s1.pdf]

A smart closed nucleic acid biosensor based on a universal endogenous reference gene detection to screen and identify mammals on site

Yuancong Xu[1], Wenjin Xiang[2], Qin Wang[3], Nan Cheng[1], Li Zhang[1], Kunlun Huang[1,2], Wentao Xu[1,2 *]

[1]Beijing Advanced Innovation Center for Food Nutrition and Human Health, College of Food Science & Nutritional Engineering, China Agricultural University, Beijing, 100083, China

[2]Laboratory of Food Safety, College of Food Science and Nutritional Engineering, China Agricultural University, Beijing 100083, China

[3]Institute of Animal Quarantine, Chinese Academy of Inspection and Quarantine, Beijing 100123, China

* To whom correspondence should be addressed: Wentao Xu; Tel/Fax: +86 010 62736479; Email: xuwentao@cau.edu.cn

Running title: A smart closed biosensor for on-site detection of mammalian DNA

Table S1

Table S1 The source of 22 animals and the NCBI Reference Sequence of *Gcg* gene

Species	Source	NCBI Reference Sequence
Pork (*Sus scrofa*)	Institute of Zoology, Chinese Academy of Sciences	XM_005671882.2
Beef (*Bos taurus*)		XM_005897283.2
Sheep (*Ovis aries*)		XM_012135657.1
Dog (*Canis lupus familiaris*)		NM_001003044.1
Rabbit (*Oryctolagus cuniculus*)		XM_008258669.2
Yat (*Bos mutus*)		XM_005897283.2
Horse (*Equus caballus*)		XM_001494268.3
Donkey (*Equus asinus*)		XM_014860143.1
Mouse (*Mus musculus*)		NM_008100.4
Monkey（*Macaca fascicularis*）		XM_005573319.2
Chicken (*Gallus gallus*)		NM_205260.4
Duck (*Anas platyrhynchos*)		XM_013106301.1
Goose (*Anser cygnoides domesticus*)		XM_013171335.1
Yellow croaker（*Pseudosciaena polyactis*）		NW_011322542.1
Trionyx（*Trionyx sinensis*）		XM_006114843.2
Bullfrog（*Rana catesbeiana*）		unknown
Sparrow（*Passer montanus*）		unknown
Water buffalo (*Bubalus bubalis*)	Tianjin Entry-Exit Inspection Bureau	XM_006073996.1
Camel (*Camelus ferus*)		XM_006176810.1
Deer (*Cervus nippon*)		unknown
Sable (*Martes zibellina*)		unknown
Human placenta（*Homo sapiens*）	Air Force General Hospital	NM_002054.4

Table S2

Table S2 The information of primers and probe in the study

Primer	Sequence（5'—3'）	Modified	Purpose	Reference
G-FIP	TGGCAGCTTGGCCTTCCAAATA**TTTT**TGAGAGACATGCTGAAGGGA	**FITC**	LAMP/LFD-LAMP	This study
G-BIP	TTGCTTGGCTGGTGAAAGGCC**TTTT**TCAACAATGGCGACCTCTTC	**BIO**		
G-F3	TTGCCAAACGTCACGATGA	——		
G-B3	TGTCTGCGGCCAAGTTCT	——		
G-SF	CAGGGCACATTCACCAGTGACT	——	Southern blot	
G-SR	ATCTCATCAGAGAAAGAACCATCAGC	——		

Table S3

Table 3 Limitation of the quantitative LAMP assay in 15 mammalian species

Species	Total sequence length(bp)	GenBank assembly accession	Limitation
Bos taurus	2,670,139,648	GCA_000003055.5	~4 copies (3.6)
Sus scrofa	2,808,525,991	GCA_000003025.4	~3 copies (3.4)
Ovis aries	2,615,516,299	GCA_000298735.2	~4 copies (3.7)
Equus caballus	2,474,929,062	GCA_000002305.1	~4 copies (3.9)
Canislupus familiaris	2,410,976,875	GCA_000002285.2	~4 copies (4.0)
Mus musculus	2,803,568,840	GCA_000001635.6	~3 copies (3.4)
Oryctolagus cuniculus	2,737,462,810	GCA_000003625.1	~4 copies (3.5)
Homo spaiens	3,232,546,710	GCA_000001405.22	~3 copies (2.9)
Bos mutus	2,645,161,911	GCA_000298355.1	~4 copies (3.6)
Equus asinus	2,391,051,217	GCA_001305755.1	~4 copies (4.0)
Macaca fascicularis	2,946,843,737	GCA_000364345.1	~3 copies (3.3)
Bubalus bubalis	2,836,166,969	GCA_000471725.1	~3 copies (3.4)
Camelus bactrianus	1,992,663,268	GCA_000767855.1	~5 copies (4.8)
Cervus		Unknown	
Martes zibellina		Unknown	

Figure S1

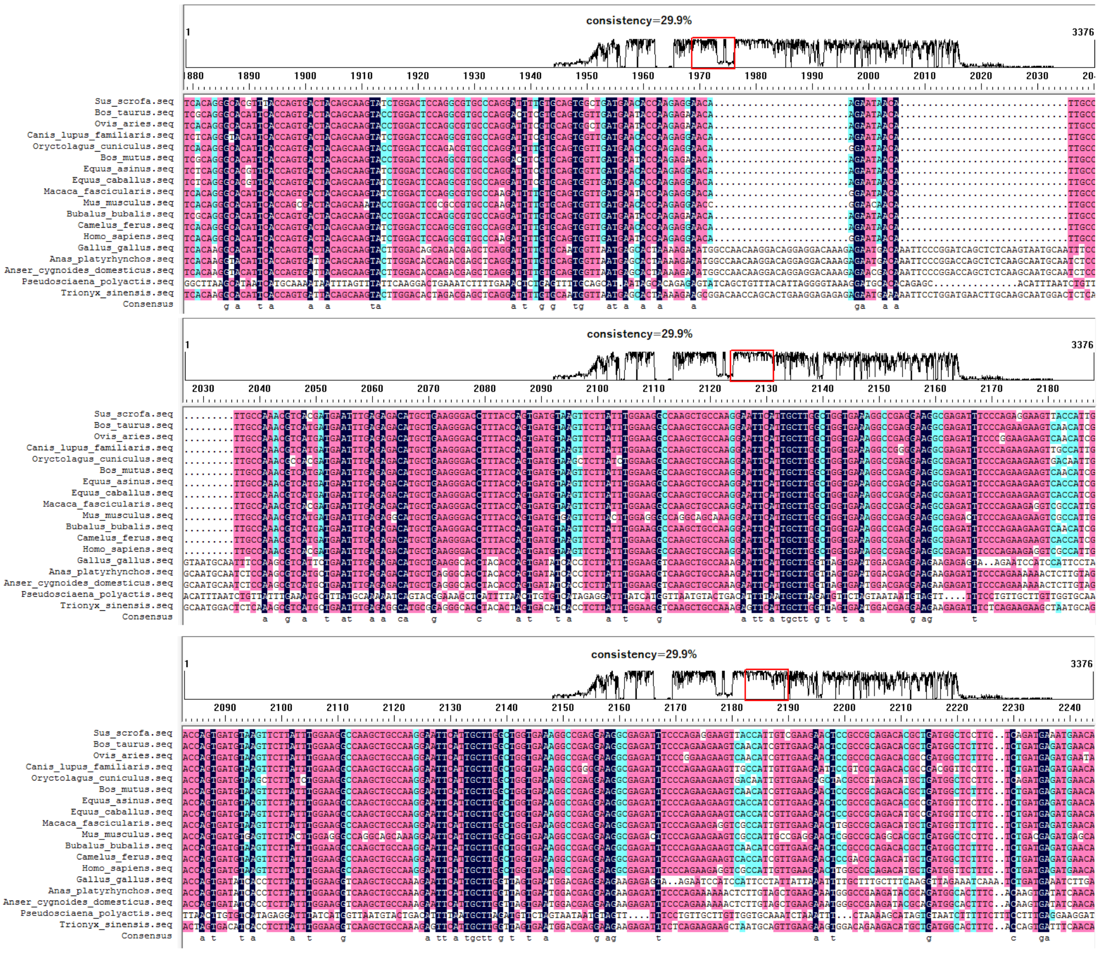

Figure S1 The DNAman V6. Multiple sequences alignment (MSA) of 13 mammalian and 5 non-mammalian Gcg sequences

Figure S2

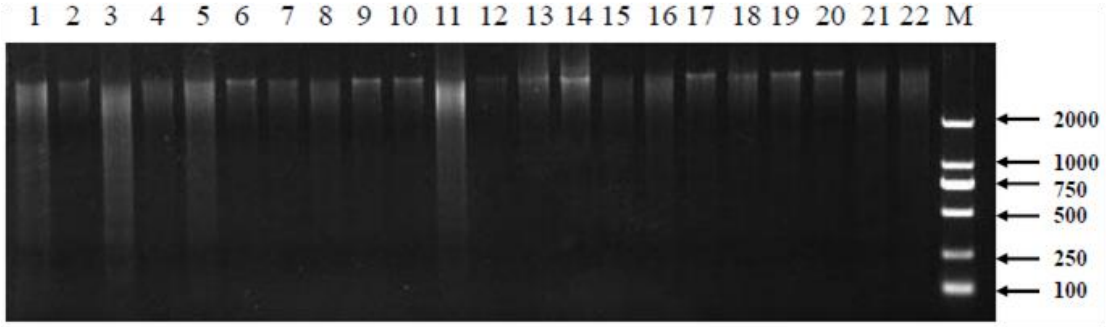

Figure S2 The agarose gel electrophoresis for genomic DNA of 22 meats. Lane 1-22: 1,donkey; 2, human; 3,deer; 4,monkey; 5,pig; 6,cattle; 7,sheep; 8,horse; 9,chicken; 10,duck; 11, goose; 12, trionyx sinensis; 13, bullfrog; 14, sparrow; 15, dog; 16, rabbit; 17, yellow croake; 18, mouse; 19, yark; 20, waterbuffalo; 21,camel; 22, sable; M: DNA Marker DL 2000

Figure S3

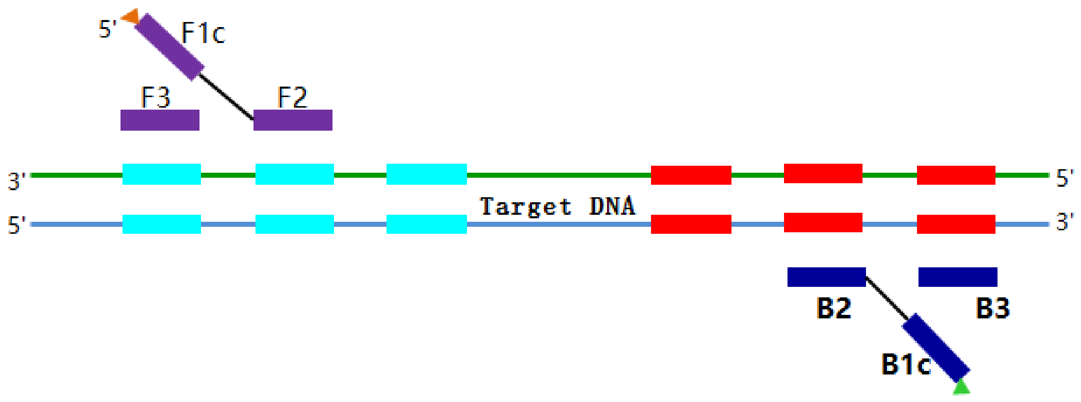


```
1      CAGGGCACAT TCACCAGTGA CTACAGCAAG TATCTGGACT CCAGGCGTGC CCAAGATTTT

61     GTGCAGTGGT TGATGAATAC CAAGAGGAAC AGGAATAACA TTGCCAAACG TCACGATGAA
                                                          F3
121    TTTGAGAGAC ATGCTGAAGG GACCTTTACC AGTGATGTAA GTTCTTATTT GGAAGGCCAA
                  F2
181    GCTGCCAAGG AATTCATTGC TTGGCTGGTG AAAGGCCGAG GAAGGCGAGA TTTCCCAGAA
        F1c                     B1
241    GAGGTCGCCA TTGTTGAAGA ACTTGGCCGC AGACATGCTG ATGGTTCTTT CTCTGATGAG
                 B2c                  B3c
301    AT
```

Figure S3 The location and sequence of the *Gcg* gene. The sequences of the primer binding sites are marked below the line.
